# ASD-GANNet: A Generative Adversarial Network-Inspired Deep Learning Approach for the Classification of Autism Brain Disorder

**DOI:** 10.3390/brainsci14080766

**Published:** 2024-07-29

**Authors:** Naseer Ahmed Khan, Xuequn Shang

**Affiliations:** School of Computer Science and Technology, Northwestern Polytechnical University, Xi’an 710072, China; naseerkhan@mail.nwpu.edu.cn

**Keywords:** cGAN, ASD, fMRI, autism, classification, multi-head attention, attention, rs-fMRI, end-to-end, augmentation

## Abstract

The classification of a pre-processed fMRI dataset using functional connectivity (FC)-based features is considered a challenging task because of the set of high-dimensional FC features and the small dataset size. To tackle this specific set of FC high-dimensional features and a small-sized dataset, we propose here a conditional Generative Adversarial Network (cGAN)-based dataset augmenter to first train the cGAN on computed connectivity features of NYU dataset and use the trained cGAN to generate synthetic connectivity features per category. After obtaining a sufficient number of connectivity features per category, a Multi-Head attention mechanism is used as a head for the classification. We name our proposed approach “ASD-GANNet”, which is end-to-end and does not require hand-crafted features, as the Multi-Head attention mechanism focuses on the features that are more relevant. Moreover, we compare our results with the six available state-of-the-art techniques from the literature. Our proposed approach results using the “NYU” site as a training set for generating a cGAN-based synthetic dataset are promising. We achieve an overall 10-fold cross-validation-based accuracy of 82%, sensitivity of 82%, and specificity of 81%, outperforming available state-of-the art approaches. A sitewise comparison of our proposed approach also outperforms the available state-of-the-art, as out of the 17 sites, our proposed approach has better results in the 10 sites.

## 1. Introduction

Autism spectrum disorder (ASD), as described by the American Psychiatric Association (APA) in the Diagnostic Statistical Manual (DSM-5) [1], is a neurological disorder characterized by a lack of communication, restrained social skills, and visible signs of repetitive behaviors, like the stacking of objects, in children who have ASD. ASD is called a “spectrum” disorder because no two people having ASD exhibit the same phenotype, as most ASD conditions vary from person to person and ASD subjects can have variations in skills, symptoms, and cognitive abilities [2]. The prevalence of ASD worldwide is around 1% [3], while estimates are even higher in high-income countries; for example, in the US [4], about 2.3% of children aged 8 years and about 2.2% of adults are diagnosed with ASD, which makes ASD a global economic burden. The early diagnosis of ASD is considered crucial because early diagnosis and the timely start of therapies and interventions in ASD subjects have shown improvement in the quality of life of people with ASD [5]. The challenging issue is that the early diagnosis of ASD is difficult because of the lack of trained ASD specialists worldwide, demographic variations across countries, and universal ASD screening tests. Most importantly, the interventions and therapies that exist around the world to target ASD are mostly designed for the adult population [6,7]. In light of this evidence, it is of significant interest to find psychological interventions, biological drugs, and artificial intelligence-based methods to tackle and treat ASD in general and find those neuro-markers and genetic factors related to ASD that can reduce the economic burden of ASD and improve the quality of lives of people suffering with ASD in particular.

### 1.1. Motivations

The functional magnetic resonance imaging (fMRI) modality has been receiving more attention from researchers in the field of neuro-science because of its non-invasiveness, high spatial resolution, and lower cost spent over time. fMRI measures the neuronal activity in the brain by measuring the magnitude of the blood-oxygenated-level-dependent (BOLD) signals [8,9]. A BOLD signal reflects the changes in blood oxygenation levels in response to neuronal activity in a brain region. The key idea of fMRI is to measure these changes, which are indicative of increased blood flow and oxygenation following neuronal activation. A range of brain disorders, including Alzheimer’s [10], attention deficit hyperactivity disorder [11], autism spectrum disorder [12], epilepsy [13], major depressive disorders [14], and Parkinson’s [15], have now been explored using fMRI, which makes fMRI a widely researched modern modality for studying various brain disorders. A plethora of fMRI studies on various brain disorders, the non-invasive nature of fMRI, and the high spatial resolution of fMRI make this imaging modality a promising research direction for pursuing and exploring brain disorders.

Of late, deep learning (DL)-based approaches [16] have revolutionized the field of artificial intelligence and are being used in a variety of tasks, such as classification, detection, segmentation, and natural language processing. The underlying idea of DL, which is also called representational learning, is to map the underlying data distribution to a non-linear latent dimension by first using a multi-layered neural network and then training the network using the back-propagation algorithm. After training, the trained neural networks are tested on unseen or testing data. DL-based approaches have shown huge potential in the fields of image classification [17], brain tumor detection [18], drug discovery [19], and natural language processing (NLP) [20], which makes DL an active area of research for many open problems, consequently motivating a community of researchers to use the ideas of DL in their respective fields. In the following, we briefly review the related articles specific to our proposed approach, with which we compare our results.

Conditional Generator Adversarial Networks (cGANs) [21] comprise an unsupervised category or class-embedded approach that is used to generate a synthetic dataset when the dataset is rare and the problem of over-fitting the model is to be reduced using the synthetically generated dataset. cGAN consists of two neural networks called the “Generator” and “Discriminator”, where the task of the Generator is to generate real data using the random vector and a class-embedding vector to fool the Discriminator, and the task of the Discriminator is to identify if the data generated by the Generator are real or fake using the real and fake data with class-embedding information. Our motivations for using the cGAN comes from its ability to generate synthetic data to reduce the problem of over-fitting because of highly dimensional connectivity features. We use the cGAN to generate connectivity features across the category of subjects so that more of the dataset on connectivity features can be generated, and our model has solved the issue of over-fitting.

### 1.2. Related State of the Art

RFEGNN [22], a “Recursive Features Extraction-based Graph Neural Network” approach, was proposed to classify ASD using spatial features extracted from recursive feature elimination and then concatenating them with the phenotype information on the subjects. The authors achieved promising results, as their fusion-based approach achieved an overall accuracy of 80%. A potential drawback of their approach is the use of a feature selection approach and a concatenated approach to fuse two sets of features. Our proposed approach neither relies on those hand-crafted features nor uses the phenotype information and still outperforms this approach, which shows the robustness of our approach.

MHSA [23] is a “Multi-Head Self Attention”-based promising approach that uses the architecture of transformer-based Multi-Head attention and a data augmentation module. The authors achieved promising results with their end-to-end approach that did not require hand-crafted features with 81.47% accuracy, 83.8% sensitivity, and 80.16% specificity. But the main weaknesses of their approach was the augment module where the authors used a sliding window approach and a large number of parameters needed for selection and experimentation. To this end, our proposed approach of cGAN-powered data augment module solves this problem by not relying on the sliding window to augment the dataset.

MVES [24] is a “Multi-View Ensemble”-based approach to tackle the classification of ASD by first extracting the mean time series of the fMRI data and then by a selection of low-/high-level functional connectivity. It features using PCA and autoencoder and finally an ensemble-based model for the classification. The authors reported a classification accuracy of 72% and a highest classification accuracy of 92.9% on the CMU site. In MVES, the authors used a fixed-length window approach to augment the data, which is also the main limitation of their approach, because by using the fixed-length window, the inherent meaningful structure of the full-length fMRI is ignored. To this end, we again firmly believe that our proposed approach is superior to their fixed-length approach because our cGAN-based data augmentation approach tackles data augmentation by generating fixed-length correlation features that do not depend on the varying time series of a subject.

NVS [25], a “Novel Features Selection”-based approach that uses a pre-trained variational autoencoder, achieved a 10-fold accuracy of 78.12% on the overall dataset. The authors also proposed an innovative activation function and devised a normalization pipeline. But their approach used the hand-crafted feature selection approach that is now considered obsolete. Our end-to-end tackles this limitation well because, it does not rely on a pre-selection of features. Moreover, the authors did not report sitewise metrics for the 17 sites, which again limits the usefulness of their approach as it might not work well on sitewise data because some sites have significantly smaller data points, which make classification a challenging task on individual sites.

MSC [26] is a “Multi-Site Clustering”-based approach that tackles the classification task using nested feature selection, where the authors first divide the dataset into two categories of ASD and HC, and then the features are selected from the two sites using single-value decomposition, resulting in an accuracy of 68.42%. The major limitations of their work are the hand-crafted features and the reporting of accuracy on fewer sites instead of all the 17 sites.

DeepGCN [27], a “Deep Graph Convolution Neural Network”-based approach, uses an end-to-end idea using Graph Convolution Network (GCN) that has achieved promising results on the ABIDE dataset and the authors have reported a cross-validated accuracy of 73.7%. The main limitation of their approach is that, although the authors used the end-to-end approach for the classification of ABIDE, they did not report a sitewise comparison of the accuracy, which limits the usefulness of their approach because the sitewise high accuracy is considered more challenging due to lesser data points, as previously discussed.

### 1.3. Contributions

The main contributions of the proposed approach are as follows:

- A cGAN-based synthetic dataset generator is proposed to augment the connectivity features of a subject. The reason for the generation of fixed-length connectivity features per subject using Pearson correlation is to avoid the variable time dimension of the subjects’ time series data.
- A Multi-Head-attention-based deep learning model is proposed here using the idea of multi-channel keys, query, and value to capture diverse aspects of the features. The motivation for this idea is because we do not want to use hand-crafted features for the classification as a Multi-Head-based attention mechanism with skip connections, tackling the task of selecting useful features in an end-to-end manner.
- A pre-trained autoencoder trained on the training subjects’ data is used to train the cGAN because we want to generate the subjects’ features which are as close to the original subjects’ distribution as possible. Therefore, when training the cGAN, in addition to the losses of the Discriminator and Generator, we also focus on the pre-trained autoencoder loss to verify if the generated subjects follow the distribution of the original datasets or not.

### 1.4. Objectives

We use correlation-based connectivity features computed using Pearson’s correlation to tackle the task of varying time series on subjects. The connectivity features have a dimension of 6670 and they pose a problem of over-fitting in the classification model because of a much smaller size of dataset. The main objectives of the proposed deep learning-based approach are to first come up with a synthetic subject’s data on ASD and HC and use an end-to-end framework for the classification of subjects into ASD and HC without using hand-crafted features. Since the ASD dataset on some sites contain very few subjects which make accurate classification very challenging, our cGAN-based data augmentation tackles this problem in an ingenious way. Moreover, The Multi-Head attention mechanism that we propose tackles the problem of over-fitting by focusing on only those areas of the brain regions which actually play their roles in the ASD, underlying neural activity patterns.

## 2. Materials and Methods

We now describe the public dataset on AN ASD-resting-state fMRI, challenges related to the dataset, and the proposed methodology in detail.

### 2.1. Dataset

Autism Brain Imaging Data Exchange (ABIDE) [28] is a public consortium that has provided the resting-state fMRI dataset on the individuals with ASD, which is pre-processed, as described in [29], where pre-processing includes steps like slice-timing correction, motion realignment, normalization, and registration. The original dataset includes 1112 individuals with 539 suffering from the ASD and 573 are typical controls, but as per the latest updates, the dataset now contains 884 total subjects as shown in Table 1. Moreover, we use Automated Anatomical Labeling (AAL) [30]-based regions of interests .

**Figure 1:**
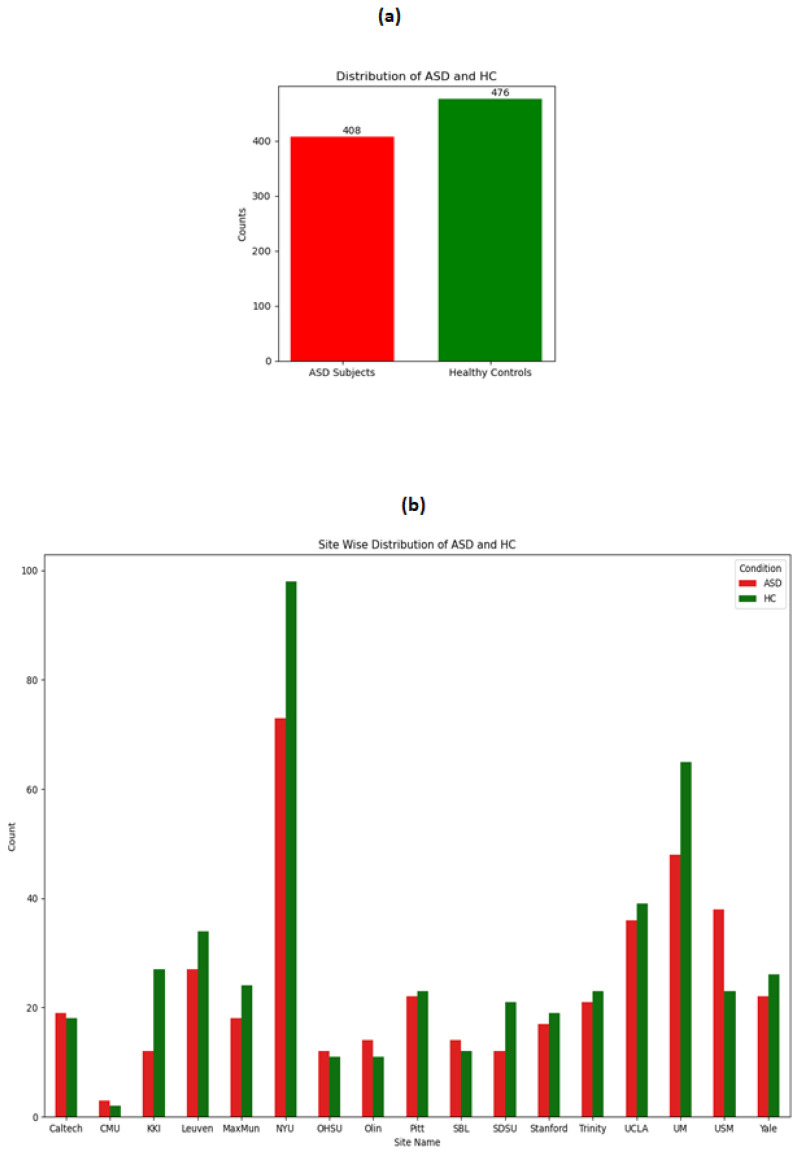
Distribution of ASD and HC: (**a**) ASD vs. HC on the combined dataset. (**b**) ASD vs. HC across 17 sites.

The distribution of ASD and HC on the whole dataset and across the sites is shown in the following Figure 1. It can be seen from the figure that although the overall distribution of ASD and HC is not imbalanced, when observing the distribution sitewise, there are drastic variations in the number of ASD and HC across sites. For example, the site CMU does not even have a total of 10 data points, whereas the NYU site has more than 100 data points. These observations make stratified split of training and testing more logical because otherwise it might be possible that no training data are included from the CMU site, leading to biases in the model.

### 2.2. Stratified Training and Testing Split

**Figure 2:**
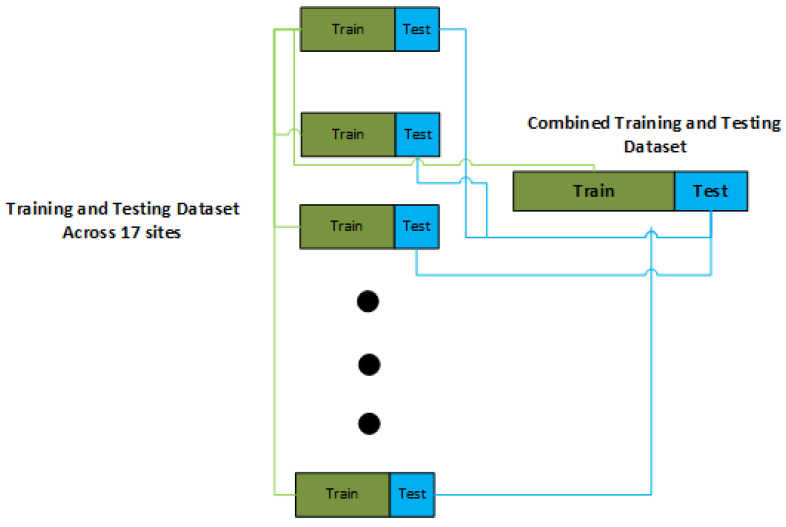
Stratified training and testing split of the dataset.

The imbalance nature of the dataset as we have just explained in the previous section necessitates us to use a stratified-based partition of the dataset into training and testing ones, as shown in Figure 2. The reason to use stratified-based splitting of dataset for training and testing is because of the varying sizes of ASD and HC across the sites. The site “SBL” has only 26 subjects while the site “NYU” has 117 subjects; so when using cross-validation-based on no-stratification, the “SBL” site might have no representation in the training set, resulting in poor performance predictions on the “SBL” testing dataset. Therefore, in the rest of study, we use a stratified-based split of dataset into the training and testing ones so that every site receives its representation in the training and testing datasets.

### 2.3. Methodology

**Figure 3:**
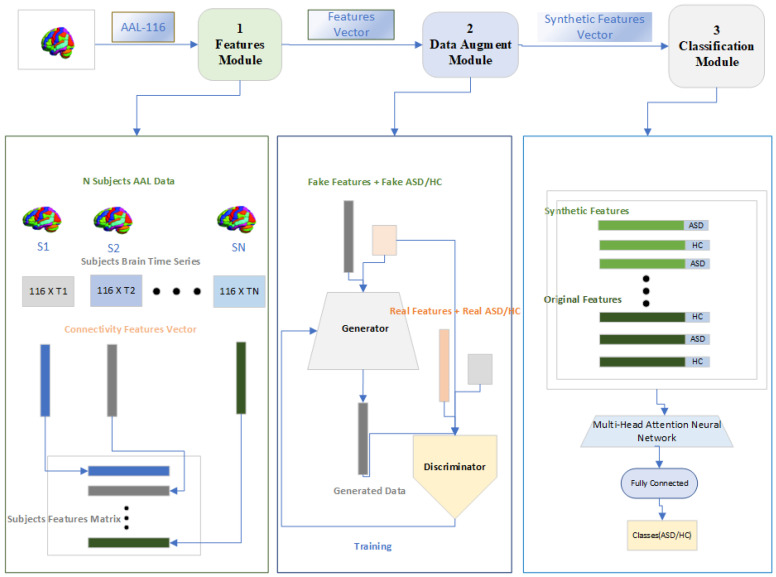
Architecture of the ASD-GANNet.

In Figure 3, we now explain our proposed model for the classification of ASD on the ABIDE pre-reprocessed rs-fMRI dataset. The proposed model consists of three modules which we call the Features Module, Data Augment Module, and a Classification Module. The general flow of the proposed approach is as follows. Firstly, the connectivity features will be calculated for both categories of subjects that is ASD and HC. Secondly, the connectivity features will be used to train a cGAN to generate synthetic connectivity features and class per subjects so that the problem of over-fitting while training can be reduced. Finally, a classification module will be used for the classification of subjects to ASD and HC using the Multi-Head attention mechanism.

In the following subsections, we briefly describe each of the three modules that are shown in Figure 3.

#### 2.3.1. Feature Module

**Figure 4:**
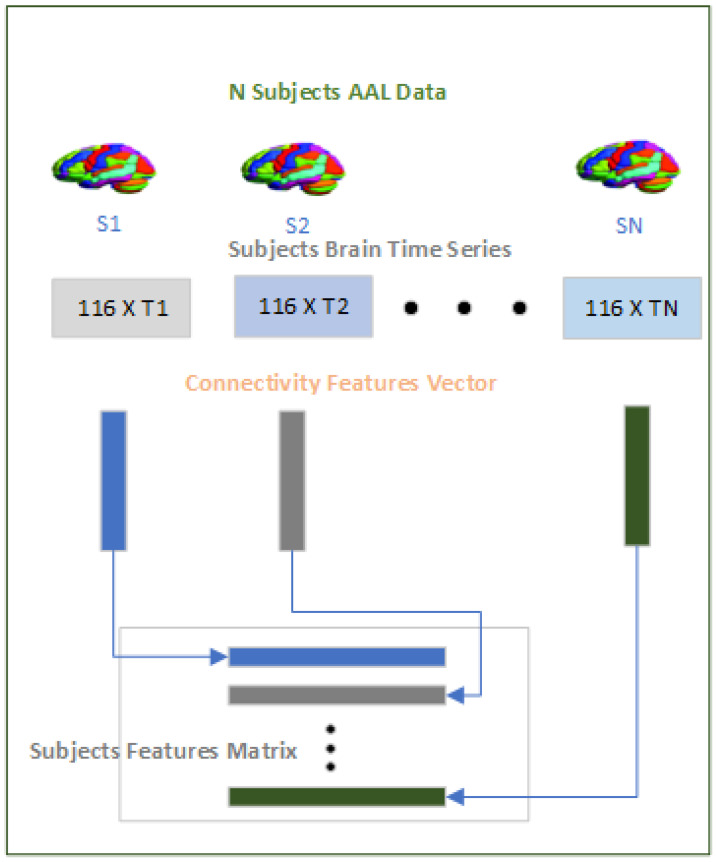
Feature module.

The purpose of the this module as shown in Figure 4 is to convert the time series data on the brain regions across subjects to a fixed-length dimension that can be processed by the “Data Augment Module” and subsequently by the “Classification Module”. The time series data on the 116 brain regions vary sometimes within site and also across sites. Tackling the varying dimension of the time series data is a significant challenge because taking a fixed-size window for the time series across subjects not only violates the inherent structure of the pre-processed fMRI but also limits generalization over the subjects. To tackle this challenge, we have used a Pearson’s correlation “*r*”, as given in Equation (1), to measure the functional connectivity between any two brain regions “*x*” and “*y*”. Since we are using the AAL-116 template in the study where each subject has 116 brain regions, the correlation will result in a symmetric 116 × 116 matrix, from which we extract the upper diagonal “6670” values excluding the diagonal, because the diagonal is always 1 as the correlation between the two same brain regions will always be 1. Therefore, for each of the subjects, whether ASD or HC, we will have a “6670” dimensional connectivity feature vector, which will be used subsequently in the rest of the modules.

(1) $$r=\frac{\sum_{i=1}^{n}\left(x_{i}-\bar{x}\right)\left(y_{i}-\bar{y}\right)}{\sqrt{\sum_{i=1}^{n}{\left(x_{i}-\bar{x}\right)}^{2}\sum_{i=1}^{n}{\left(y_{i}-\bar{y}\right)}^{2}}}$$

The motivations of using the resting-state functional connectivity (rs-fMRI) features is deeply rooted in how the neuronal activity in a disordered brain takes place as it has been shown that the resting state brain of a subject, when the subject does not perform any task, is different from the neuronal activity of the subjects without a disorder [31].

#### 2.3.2. Data Augment Module

**Figure 5:**
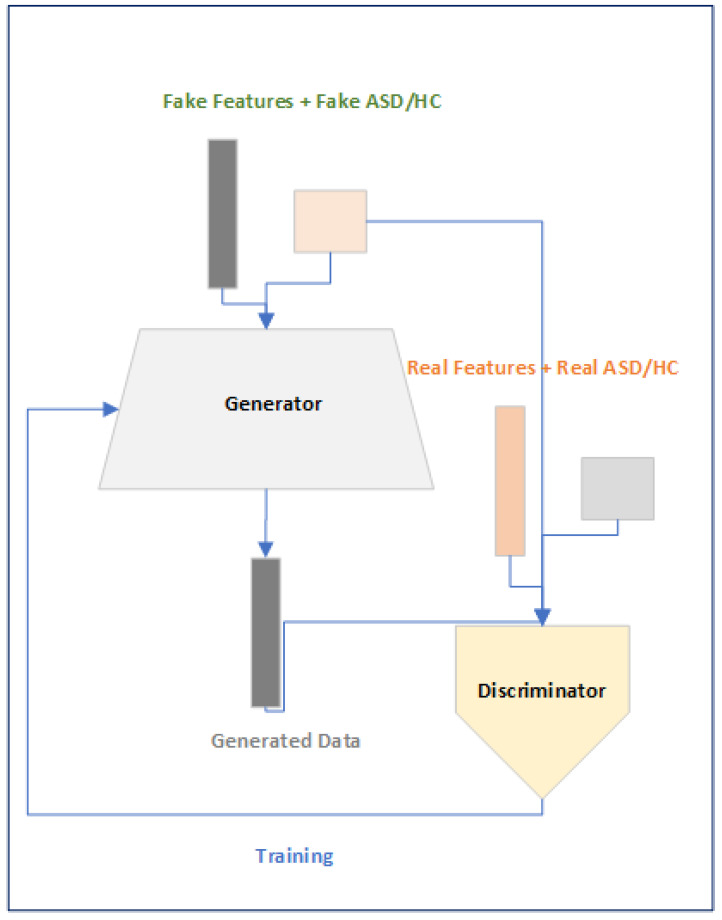
Data Augment Module.

The purpose of this module, as shown in Figure 5, is to generate synthetic 6670 dimensional features across ASD and HC because the size of the dataset when compared to the size of features dimension is a lot lower, leading to the over-fitting of the deep learning models. We use the cGAN-based approach [21] to generate synthetic features across subjects as we not only want the features but we also want to condition the features across the subjects based on the subjects’ conditions, whether belonging to ASD or HC. The objective function of the conditional GAN (cGAN) as explained in [32] is given in Equation (2), where “*G*” and “*D*” are the Generator and Discriminator, “*x*” is the features vector, “*y*” is the class of the subject and “*z*” is the random vector. The main purpose of the cGAN min–max approach is to fool the Discriminator over time by the generator by generating subjects as close to the real data.

(2) $$\min_{G}\max_{D}V\left(D,G\right)=E_{x∼p_{data}\left(x\right)}\left[\log D\left(x|y\right)\right]+E_{z∼p_{z}\left(z\right)}\left[\log\left(1-D\left(G\left(z|y\right)\right)\right)\right]$$

The motivations of using the cGAN-based approach is to overcome the problem of using fixed-size subject time series data and to minimize the problem of over-fitting that is faced when training the DL model.

#### 2.3.3. Classification Module

**Figure 6:**
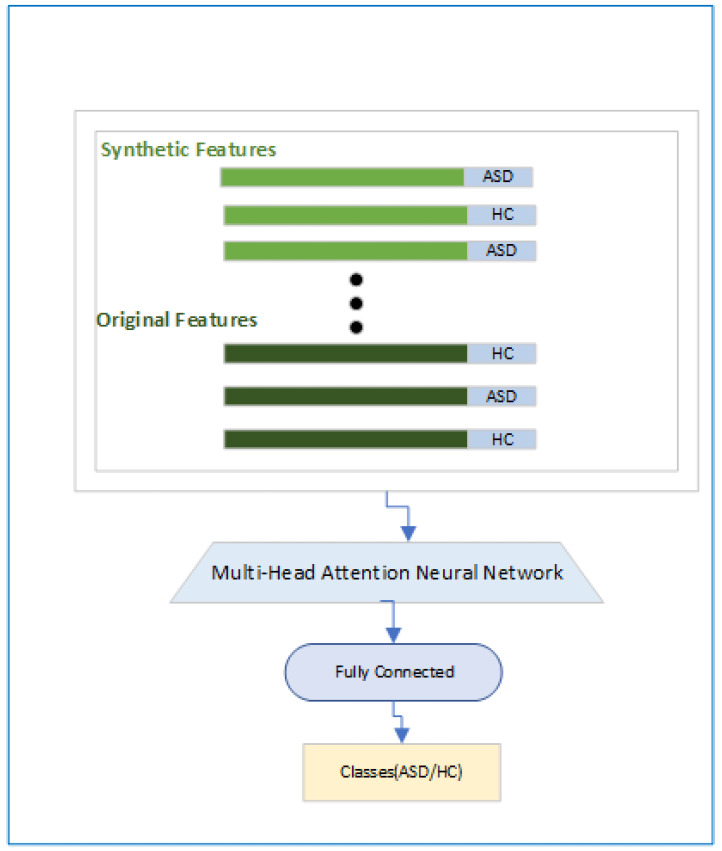
Classification module.

In this module, as shown in Figure 6, we show how the final objective of the classification will be achieved. Here, first, we will have features and associated labels for the original and synthetic ASD and HC subjects, and then a Multi-Head attention-based module [33] will be used so that focus is on the relevant features, followed by a fully connected layer and a classification head.The Multi-Head attention-based module is designed using Equation (3), where “*Q*”, “*K*” and “*V*” are called the query, key, and value and “*d*” is a normalization constant.

(3) $$Attention\left(Q,K,V\right)=softmax\left(\frac{QK^{T}}{\sqrt{d_{k}}}\right)V$$

#### 2.3.4. Algorithm

In the following section, we explain the proposed approach using a pseudo-algorithm as written in Algorithm 1.

The proposed algorithm takes an input of the subject data with an associated label on the training dataset and outputs the accuracy of the model on the testing dataset. Algorithm 1 is divided into the three submodules: Feature Module, Data Augment Module, and the Classification Module. The function “Train-Test-Split” as described in line 3 is used to partition the dataset into the training and testing datasets using stratification. The function “PearsonCorr” is the correlation coefficient as mentioned in Equation (1) and the function “UpperTria” is used to extract the unique values from the symmetric 116 × 116 connectivity matrix. The function “Generator” and “Discriminator” are the neural networks used to train the cGAN in an adversarial manner. The function “Multi-Head”, “FC’s”, and “Class” are the Multi-Head-based attention layer, fully connected layers, and the classification layer of the classification module.
**Algorithm 1** Proposed cGAN-Powered Multi-Head Attention Approach’s Algorithm1:**Input** ← D, **Output** ← {}, cGANEpochs, synthEpochs, classEpochs2:*X,Y* ← *D* (*X* ← Features Matrix, *Y* = ASD/HC)3:$X_{train}$, $X_{test}$, $Y_{train}$, $Y_{test}$ ← Train-Test-Split(X,Y)4:$F_{train}$ ← {}, $F_{test}$ ← {}5:**(Feature Module):**6:**for** $x_{train}$, $x_{test}$, $y_{train}$, $y_{test}$ in ($X_{train}$,$X_{test}$,$T_{train}$,$Y_{test}$) **do**7:      $subjectTrain_{116xT}$ =$x_{train}$, $subjectTest_{116xT}$ =$x_{test}$8:      $Train_{116\times 116}$ ← *PearsonCorr*($subjectTrain_{116xT}$)9:      $Test_{116\times 116}$ ← *PearsonCorr*($subjectTest_{116xT}$)10:    $Train_{6670}$ ← *UpperTria*($Train_{116\times 116}$)11:    $Test_{6670}$ ← *UpperTria*($Test_{116\times 116}$)12:    $F_{train}$ ← $F_{train}$U { $Train_{6670}$, $y_{train}$ }13:    $F_{test}$ ← $F_{test}$U { $Test_{6670}$, $y_{test}$ }14:**end for**15:**(Data Augment Module):**16:$F_{synthetic}$ ← {}17:**for** epoch in cGANEpochs **do**18:    $Data_{random}$,$label_{random}$ ← *Random()*19:     $F_{real}$,$Y_{real}$ ← *Generator*($Data_{random}$,$label_{random}$)20:    $F_{real}$,$Y_{real}$ ← *Subset($F_{train}$)*21:    *Discriminator($F_{fake}$,$Y_{fake}$)*22:    *Discriminator($F_{real}$,$Y_{real}$)*23:**end for**24:**for** epoch in synthEpochs **do**25:     $D_{synthetic}$,$label_{synthetic}$ ← *Generator*()26:    $F_{synthetic}$ ← $F_{synthetic}$U { $D_{synthetic}$, $label_{synthetic}$ }27:**end for**28:**(Classification Module):**29:**for** epoch in classEpochs **do**30:     $F_{1}$ ← *Multi-Head($F_{train}$,$F_{synthetic}$)*31:     $F_{2}$ ← *${FC}_{1}$($F_{1}$)*32:     $F_{3}$ ← *${FC}_{2}$($F_{2}$)*33:     $F_{4}$ ← *${FC}_{3}$($F_{3}$)*34:     $Model_{trained}$ ← *Class($F_{4}$)*35:**end for**36:**Output** ← $Model_{trained}$($F_{test}$)

## 3. Results

We now describe experimental settings related to the autoencoder, cGAN, and Multi-Head attention and the types of experiments related to the combined site and sitewise that we performed in the current study.

### 3.1. Experimental Settings

We use a “Denoising Autoencoder” to reconstruct connectivity features by mapping the original data into a latent representation. Then, we generate connectivity features using the cGAN, and finally, validate the generated subjects’ data distribution with the original subjects’ feature data by mapping the generated subjects’ features to the latent representation.

#### 3.1.1. Denoising Autoencoder

Denoising Autoencoder has five encoder layers of size 4000, 3000, 2000, 1000, and 500 and five decoder layers of size 1000, 2000, 3000, 4000, and 6670. “*Batch Normalization*” and “*Relu*” activation are used after each of the encoder and decoder layers. “*Learning Rate*” was set to 0.00001, “*Epochs*” set to 5000, “*Batch Size*” set to 64 and the “*Adam*” optimization was used for the training of training while a random “*Gaussian*” noise was added to the input to make the decoder part of the model more robust.

#### 3.1.2. cGAN

The Generator has a total of five layers, the noise dimension for *random vector* generation is set to 500, and the *condition dimension* for ASD and HC is also set to 500 as our purpose is to train the conditional cGAN. Before the first layer, we used the *embedding layer* for converting the two categories to a combined noise and condition layer, resulting in 1000 dimension. After the embedding layer, the layers have sizes of 2000, 3000, 4000, 5000, and 6670, where each layer is followed by the *spectral normalization* layer, the *batch normalization* layer, and a *leakyRelu* activation of 0.1. The Discriminator has five layers of sizes 4000, 2000, 1000, 500, and 1, where each layer is followed by spectral normalization and a *LeakyRelu* of 0.1. Before the first layer of the Discriminator, the *embedding layer* is used to convert the ASD/HC categories to 500 *conditional dimension* and then the first input layer has an input size of the *feature size*, that is 6670, and a *conditional dimension* size of 500. The *learning rate* for the Discriminator and Generator was set to 0.00001, optimization was set to *Adam*, *batch size* to 64, and the total *epochs* were set to 5000 for the training of the conditional cGAN.

#### 3.1.3. Classification

The classification module consisted of a Multi-Head attention block, which has an input of size 6670, followed by a *Multi-Head attention* layer with 10 heads, a skip layer from input to the output of the Multi-Head attention layer, and a *normalization layer* followed by three *feed forward layers* of size 3000, 2000, and 1000, 1 containing skip connections from previous layer and a normalization layer. We set *epochs* to 5000, *batch size* to 64, *learning rate* to 0.0001, and optimizer to *Adam* for the training of the classification module.

### 3.2. Experiments

There are two kinds of experiments that we conducted for the current work. The first was an overall accuracy comparison where the whole dataset was first divided into the training and testing split using stratification. The second was training the model on the combined training data and then testing it on the combined testing dataset. A sitewise comparison, where we used the largest site NYU for the training, trained the model on this site and then tested on the remaining sites, but excluding the *CMU* site because it contained too few subjects to compare.

#### 3.2.1. Overall Comparison

**Figure 7:**
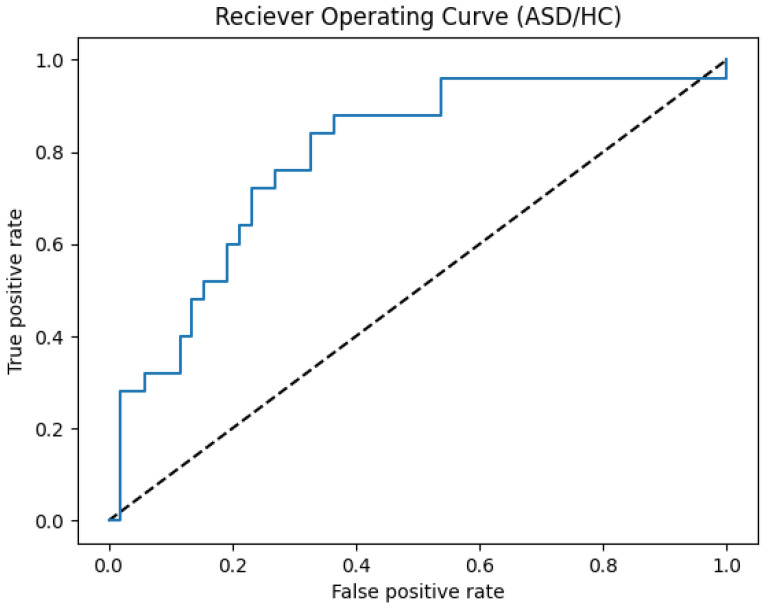
ROC curve on the combined dataset.

In the first experiment, we compare the results of the proposed approach with the state-of-the-art approaches as shown in Table 2. It can be seen that our proposed approach accuracy, precision, and recall have outperformed the other approaches, which validates the effectiveness of the proposed approach on the whole data. To analyze the classification model performance, the Receiver Operating Curve (ROC) is performed by varying the thresholds of prediction based on the combined dataset, as shown in Figure 7.

#### 3.2.2. Sitewise Comparison

In the second comparison, we used the “NYU” training total dataset as the training dataset and tested the trained model on the rest of the 15 sites excluding the “CMU” site, as performed in [23]. We have shown the results of the proposed approach with the state-of-the-art approaches and it can be seen that our proposed approach has outperformed the other approaches in 10 out of the 15 sites, validating the effectiveness of the proposed approach (Table 3).

## 4. Discussion

**Figure 8:**
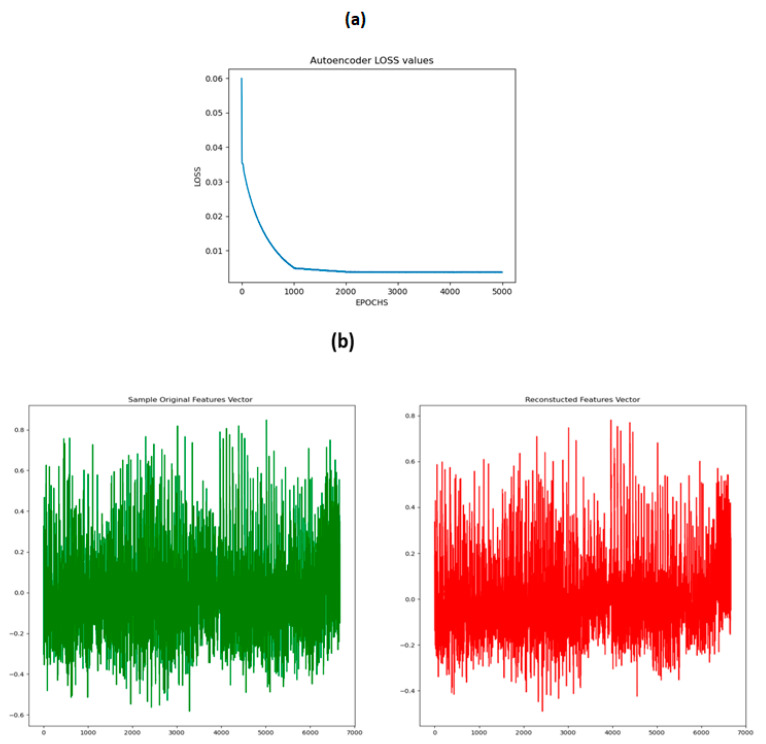
Training of the Denoising Autoencoder (**a**). Loss of the Denoising Autoencoder model. (**b**) Distribution of the reconstructed and the original features.

The generation of the synthetic subject’s connectivity features using cGAN is not a trivial task because we have no prior validation method about the goodness of the synthetic subject’s feature set. To tackle this issue, we sampled a small subset of the training data and trained a Denoising Autoencoder on the training dataset. The objective was that after training the Denoising Autoencoder on a small subset of subjects, it will learn the latent representation of the training dataset. Secondly, during the cGAN training stage, the generated subjects will be fed to the trained Denoising Autoencoder and then the reconstructed subject using the decoder part will be compared to the original input to measure the loss. This step is crucial because even after the minimization of the loss of the Generator and the Discriminator of the cGAN, we should know how close the generated subject is to the distribution of the original subjects. The Denoising Autoencoder loss and the comparison of the original and the reconstructed features are shown in Figure 8.

**Figure 9:**
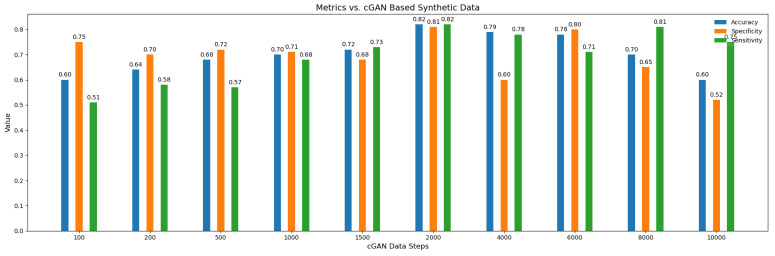
Accuracy, sensitivity, and specificity metric comparison with the cGAN generated data.

We have carried out experimentation with a varying number of synthetic subjects as shown in Figure 9. It can be seen that the results improve when the number of the synthetic subjects are 2000. A pattern can be seen in the values of the accuracy across the synthetic subjects, when the number of synthetic subjects are fewer, and the value of accuracy is low, but it gradually increases and peaks when the number of subjects are 2000, which shows that the over-fitting issue is gradually resolved by the model when attaining a sufficient number of subjects. On the other hand, too many synthetic subjects is also not a favorable scenario for the model because, in that case, the model will learn more and more the distribution of synthetic data instead of the original data and will forget about the true distribution, resulting in lower accuracy.

### Ablation Studies

We have also compared the performance of our model by taking out the essential components from our proposed approach to analyze how the proposed model behaves in scenarios when those components are present or absent in the model. For the purpose of this comparison, we performed two kinds of experiments where we first only used the cGAN component, named as “cGAN Only”, in which the downstream task of classification was performed using a simple neural network consisting of seven fully connected layers of 4000, 3000, 2000, 1000, 500, 50, and 1, where each layer was followed by “BatchNorm”, “ReLU”, and “Dropout” of 10%. In the second experiment, we only used the “Muti-Head” attention layer named as “Multi-Head Only” and did not use cGAN for synthetic data generation.

For both kinds of experiments, the results of cross-validated accuracy on the whole dataset and sitewise are displayed in Table 4 and Table 5, respectively. It is obvious from both the tables that the accuracy results suffered significantly in both the comparison, which proves that both the cGAN and Multi-Head attention component are essential for the proposed approach.

## 5. Conclusions

In this paper, we proposed ASD-GANNet, a cGAN-based data augmentation technique for the classification of ASD on the fMRI pre-processed ABIDE dataset. The challenge of synthetic data generation in the presence of varying time points across subjects was tackled by generating the fixed-length connectivity features as connectivity features across subjects have a fixed dimension. Our proposed framework is promising as we not only achieved good performance but also compared our results with the state-of-the-art methods in the literature and proved the effectiveness of the proposed approach.

### Limitations

The major limitation of the proposed approach is that we were not able to generate the original time-varying synthetic data using cGAN. There are varying time points across the sites per subject and it is very challenging to condition the cGAN on this additional time condition. In future work, we will explore how the subjects belonging to ASD or HC could be generated instead of the fixed-length correlation features and how the synthetic subjects’ distributions align with the overall original data distribution and the sitewise distribution on the ASD and HC subjects.

## Figures and Tables

**Table 1:** Pre-processed rs-fMRI dataset provided by ABIDE.

	Site Name	ASD ^1^	HC ^2^	Total
1	CALTECH	19	18	37
2	CMU	3	2	5
3	KKU	12	27	39
4	LEUVEN	27	34	61
5	MAXMUN	18	24	42
6	NYU	73	98	171
7	OHSU	12	11	23
8	OLIN	14	11	25
9	PITT	22	23	45
10	SBL	14	12	26
11	SDSU	12	21	33
12	STANFORD	17	19	36
13	TRINITY	21	23	44
14	UCLA	36	39	75
15	UM	48	65	113
16	USM	38	23	61
17	YALE	22	26	48
	**TOTAL**	**408**	**476**	**884**

^1^ ASD = Autism spectrum disorder, ^2^ HC = healthy controls.

**Table 2:** Tenfold cross-validation based on overall dataset comparison with the state of the art (%).

	Method	Accuracy	Precision	Recall	Sensitivity	Specificity
1	RFEGNN [22]	80.63	80.21	-	76.24	-
3	MHSA [23]	81.40	83.80	80.16	83.80	80.16
4	MVES [24]	72.00	-	-	-	-
5	NVS [25]	78.00	-	-	80.00	80.19
6	MSC [26]	68.42	-	-	70.05	63.64
7	DeepGCN [27]	73.02	72.97	68.80	-	-
8	**ASD-GANNet**	**82.00**	**84.00**	**81.00**	82.00	81.00

**Table 3:** Site wise dataset comparison with the state-of-the-art approaches (%).

	Site	Size	MHSA [23]	MVES [24]	ASD-GANNet
1	CALTECH	37	64.60	71.40	**71.60**
2	KKI	39	79.60	75.00	75.40
3	LEUVEN	61	70.40	72.10	**73.20**
4	MaxMun	42	66.40	61.50	**66.90**
5	OUSH	23	66.00	-	60.00
6	Olin	25	76.00	73.50	**76.00**
7	Pitt	45	65.80	74.50	73.00
8	SBL	26	89.30	64.30	65.00
9	SDSU	33	75.70	79.30	**79.70**
10	Stanford	36	77.50	69.20	70.00
11	Trinity	44	73.10	73.30	**74.00**
12	UCLA	75	69.30	77.60	**78.50**
13	USM	113	76.90	87.30	**88.10**
14	UM	61	74.90	75.70	**75.90**
15	Yale	48	75.10	80.00	**81.10**

**Table 4:** Tenfold cross-validation-based overall dataset comparison with reduced model’s component (%).

	Method	Accuracy	Precision	Recall	Sensitivity	Specificity
1	cGAN Only	66.10	68.50	68.20	69.50	55.30
2	Multi-Head Only	65.00	60.30	59.60	55.50	60.00
3	**ASD-GANNet (GAN + Multi-Head)**	**82.00**	**84.00**	**81.00**	82.00	81.00

**Table 5:** Sitewise dataset comparison with model’s reduced component (%).

	Site	GAN Only	Multi-Head Only	ASD-ccGANNet (GAN + Multi-Head)
1	CALTECH	59.20	50.00	**71.60**
2	KKI	50.40	36.00	75.40
3	LEUVEN	48.00	30.00	**73.20**
4	MaxMun	44.00	55.60	**66.90**
5	OUSH	50.00	55.30	60.00
6	Olin	59.00	58.00	**76.00**
7	Pitt	48.50	51.50	73.00
8	SBL	44.00	49.00	65.00
9	SDSU	59.60	59.00	**79.60**
10	Stanford	58.06	50.55	70.00
11	Trinity	59.60	50.60	**74.00**
12	UCLA	58.12	58.90	**78.50**
13	USM	50.14	50.55	**88.10**
14	UM	60.13	61.60	**75.90**
15	Yale	59.36	50.15	**81.10**

## Data Availability

ABIDE pre-processed fMRI dataset is a publicly available dataset maintained at http://preprocessed-connectomes-project.org/abide/ (accessed on 1 May 2024). To download the dataset, users can go to the Github tab of the mentioned url and under the “abide” repo can run the script named “download_abide_preproc.py” to download all the pre-processed fMRI datasets of ASD.

## Abbreviations

The following abbreviations are used in this manuscript:

ABIDE	Autism Brain Imaging Data Exchange
ASD	Autism Spectrum Disorder
cGAN	Conditional Generative Adversarial Networks
fMRI	Functional Magnetic Resonance Imaging
GAN	Generative Adversarial Networks
WGAN	Wasserstein Generative Adversarial Networks
rs-fMRI	Resting State Functional Magnetic Resonance Imaging
